# Identification and functional assay of the interaction motifs in the partner protein OsNAR2.1 of the two-component system for high-affinity nitrate transport

**DOI:** 10.1111/nph.12986

**Published:** 2014-08-07

**Authors:** Xiaoqin Liu, Daimin Huang, Jinyuan Tao, Anthony J Miller, Xiaorong Fan, Guohua Xu

**Affiliations:** 1 State Key Laboratory of Crop Genetics and Germplasm Enhancement, Nanjing Agricultural UniversityNanjing, 210095, China; 2 MOA Key Laboratory of Plant Nutrition and Fertilization in Low-Middle Reaches of the Yangtze River, Nanjing Agricultural UniversityNanjing, 210095, China; 3 Metabolic Biology Department, John Innes CentreNorwich Research Park, Norwich, NR4 7UH, UK

**Keywords:** high-affinity transport system two-component transporter, interaction motifs, NAR2.1, NRT2.3a, *Oryza sativa*

## Abstract

- A partner protein, NAR2, is essential for high-affinity nitrate transport of the NRT2 protein in plants. However, the NAR2 motifs that interact with NRT2s for their plasma membrane (PM) localization and nitrate transporter activity have not been functionally characterized.
- In this study, OsNAR2.1 mutations with different carbon (C)-terminal deletions and nine different point mutations in the conserved regions of NAR2 homologs in plants were generated to explore the essential motifs involved in the interaction with OsNRT2.3a.
- Screening using the membrane yeast two-hybrid system and *Xenopus* oocytes for nitrogen-15 (^15^N) uptake demonstrated that either R100G or D109N point mutations impaired the OsNAR2.1 interaction with OsNRT2.3a. Western blotting and visualization using green fluorescent protein fused to either the N- or C-terminus of *OsNAR2.1* indicated that OsNAR2.1 is expressed in both the PM and cytoplasm. The split-yellow fluorescent protein (YFP)/BiFC analyses indicated that OsNRT2.3a was targeted to the PM in the presence of OsNAR2.1, while either R100G or D109N mutation resulted in the loss of OsNRT2.3a-YFP signal in the PM.
- Based on these results, arginine 100 and aspartic acid 109 of the OsNAR2.1 protein are key amino acids in the interaction with OsNRT2.3a, and their interaction occurs in the PM but not cytoplasm.

## Introduction

The high-affinity transport system (HATS) for nitrate uptake in algae and plant is mediated by a two*-*component NRT2*/*NAR2 transport system, and some high-affinity NRT2 transporters require the partner protein, NAR2, to function (Quesada *et al*., 1994; Zhou *et al*., 2000; Tong *et al*., 2005; Araki & Hasegawa, 2006; Miller *et al*., 2007; Cai *et al*., 2008; Plett *et al*., 2010; Feng *et al*., 2011a,b; Yan *et al*., 2011). Further support for a two-component high-affinity nitrate influx system was confirmed using *Xenopus* oocyte expression analysis. Compared to water-injected *Xenopus* oocytes, *CrNAR2* increased nitrate transport when co-expressed with *CrNRT2.1* mRNA (Zhou *et al*., 2000). Similar results were reported for barley genes, which only transported nitrates when co-injected with both *HvNRT2.1* and *HvNAR2.3* mRNAs in *Xenopus* oocytes (Tong *et al*., 2005). The barley proteins were also investigated, and both were nitrate-inducible and co-localized in the root plasma membrane fraction (Ishikawa *et al*., 2009). In *Arabidopsis*, the interactions between AtNRT2.1 and AtNAR2.1 proteins were examined using yeast split ubiquitin and oocyte expression systems, and the results suggested that functional high-affinity nitrate transport may involve an interaction between AtNRT2.1 and AtNAR2.1 (Orsel *et al*., 2006). However, not all members of the *AtNRT2* family require a second gene product for functional nitrate uptake (Chopin *et al*., 2007; Kotur *et al*., 2012). All *NRT2s*, excluding *AtNRT2.7*, restored growth and β-galactosidase activity in the yeast split ubiquitin system and split yellow fluorescent protein (YFP) fluorescence in *Arabidopsis thaliana* protoplasts when co-expressed with AtNAR2.1 (Kotur *et al*., 2012). It was recently reported that OsNAR2.1 is a partner in a two-component nitrate transport system with OsNRT2.1/OsNRT2.2 in rice (*Oryza sativa*) and OsNRT2.3a in the *Xenopus* oocyte and yeast-two hybrid system (Feng *et al*., 2011a,b; Yan *et al*., 2011).

In barley, both *HvNRT2.1* and *HvNAR2.3* localized in the plasma membrane (PM) (Wirth *et al*., 2007), and HvNRT2 and HvNAR2 proteins co-localized in the PM of barley roots (Ishikawa *et al*., 2009). *AtNRT2.1* is mainly localized in the PM of root cortical and epidermal cells, and is involved in the expression of *AtNAR2.1* in the PM (Chopin *et al*., 2007; Wirth *et al*., 2007). In *Arabidopsis* protoplasts, NRT2.1 and NAR2.1 polypeptides interact directly at the PM (Yong *et al*., 2010). Kotur *et al*. (2012) reported that, with the exception of AtNRT2.7, all remaining NRT2 transporters interacted strongly with AtNAR2.1, mostly at the PM. In rice, Tang *et al*. (2012) reported that OsNRT2.3a is a PM protein.

A study on the interaction motif in the two-component system using affinity column binding analysis and recombinant proteins was suggestive of a role of the carbon (C)-terminus of HvNRT2.1 in binding to the central region of HvNAR2.3 (Ishikawa *et al*., 2009). Furthermore, Kawachi *et al*. (2006) reported that the replacement of Asp105 in the middle region of *AtNAR2.1* significantly reduced nitrate uptake and accumulation in *Arabidopsis* plants.

Although a K(2)K(2)LCY(2)S(3)RxWR(3)D(4)DK motif defining the NAR2 family was originally identified (Tong *et al*., 2005), the motifs of NAR2.1 proteins that interact with NRT2 proteins for their PM localization and nitrate transporter activity have not been functionally characterized. Here, we used the yeast two-hybrid system, *Xenopus* oocyte nitrogen-15 (^15^N) uptake experiments, and the split YFP-labeling system in rice blade protoplasts to examine the interaction between OsNAR2.1 and OsNRT2.3a. To identify the interaction motif of OsNAR2.1, we generated different C-terminal deletions and point mutations in the central region of OsNAR2.1. Green fluorescent protein (GFP) fused to the N- and C-terminus of OsNAR2.1 was used to determine the localization of *OsNAR2.1* expression in rice cells.

## Materials and Methods

### Mutation of *OsNAR2.1*

OsNAR2.1 is a protein of 206 amino acids (AA) with the open reading frame (ORF) *OsNAR2.1* (621 bp). We generated two deletions at the C-terminal end of OsNAR2.1 from AA 201–206 (597 bp) and from AA 180–206 (534 bp). The primers used to create the AA201–206 and AA180–206 deletions are listed in Supporting Information Table S1.

The highly conserved amino acids of NAR2s, including OsNAR2.1, AtNAR2.1, HvNAR2.3, CrNAR2, OsNAR2.2, HvNAR2.1, HvNAR2.2, TaNAR2.1 and ZmNAR2.1, where point mutations were generated (W66G, V85F, C88G, R100G/R100K, K101F, D109N, R144G, A150G and G158R, respectively) in the central region of OsNAR2.1 are shown in Supporting Information Fig. S1(a,b). The original *OsNAR2.1* in the pBT3-C construct was generated as described previously (Yan *et al*., 2011). Reverse PCR based on *OsNAR2.1* in the pBT3-C construct was used for point mutation construction, and the primers used are listed in Supporting Information Table S1.

### Split ubiquitin protein–protein interaction analysis

Protein–protein interactions were examined using a DUAL membrane pairwise interaction kit (Dualsystems Biotech AG, Schlieren, Switzerland) (Yan *et al*., 2011). Full-length cDNAs of the *OsNAR2.1* and *OsNAR2.1* mutations were cloned in-frame with the Nub sub-domain of ubiquitin into pBT3-C (LEU2: L, KanR) expression vectors. The primers for subcloning of the C-terminal deletion or point-mutated *OsNAR2.1* into pBT3-C *OsNAR2.1* are listed in Supporting Information Table S1. The *OsNRT2.3a* cDNA was cloned in-frame with the Cub sub-domain of ubiquitin in pPR3-N (TRP1: W, AmpR) expression vectors using primers were described previously (Yan *et al*., 2011). Point mutations of OsNAR2.1 were created at the base of *OsNAR2.1* in pBT3-C and all OsNAR2.1 point and deletion mutations in pBT3-C were respectively co-introduced with *OsNRT2.3a* in pPR3-N into the yeast strain NMY51. Two reporter genes (His and Ade: H and A) allowed the yeast to grow on selective medium (SD-AHLW), after which β-galactosidase (Lac Z) activity assays were performed (Yan *et al*., 2011).

### Functional assay of *OsNAR2.1* and *OsNRT2.3a* in *Xenopus* oocytes

mRNA synthesis of *OsNAR2.1* with point mutations and *OsNRT2.3a* cDNAs, as well as analyses of ^15^N–NO_3_^−^ uptake in oocytes were performed as described previously (Yan *et al*., 2011). *OsNAR2.1* and *OsNRT2.3a* were codon optimized and synthesized by Genescript Company (Nanjing, China). cDNA optimization for *Xenopus* oocyte expression and subcloning into pT7Ts, oocyte preparation, mRNA injection, and ^15^N–NO_3_^−^ uptake assays were conducted as described previously (Feng *et al*., 2013). Oocytes injected with the genes and water were incubated for 16 h in MBS containing 0.5 mM ^15^N–NO_3_^−^. Primers for gene subcloning into pT7Ts are listed in Supporting Information Table S2.

### Constructs for the BiFC assay

OsNRT2.3a and OsNAR2.1 were tagged with the N- and C-terminal halves of EYFP using pSAT vectors (Citovsky *et al*., 2008). Full-length coding sequence for *OsNAR2.1* was fused in-frame with the N-terminal half of EYFP in the PSAT1-nEYFP-C1 vector (nEYFP-OsNAR2.1), which included residues 1–174 of EYFP. The *OsNRT2.3a* cDNA was fused in-frame to the C-terminal half of EYFP in PSAT1-cEYFP-N1 (OsNRT2.3a-cEYFP), which included residues 175–240 of EYFP. The primers used are listed in Supporting Information Table S3. Plasmid pairs were introduced into rice blade protoplasts using the methods described previously (Tang *et al*., 2012). Co-transfected PSAT1-nEYFP-C1 and PSAT1-cEYFP-N1 was used as a control for YFP fluorescence assays.

### Constructs for transient expression of *OsNAR2.1*

To examine subcellular localization of *OsNAR2.1*, we constructed N-terminal GFP fusions (GFP-OsNAR2.1) and C-terminal GFP (OsNAR2.1-GFP) driven by the cauliflower mosaic virus 35S promoter and transfected the derived expression vector into rice protoplast and tobacco epidermis cells.

The expression construct of GFP-OsNAR2.1 (35S:GFP-NAR2.1) was generated using pSAT6-EGFP-C1 (Tzfira *et al*., 2005) and pRCS2-ocs-nptII expression vectors (Goodin *et al*., 2007). The ORF of wild-type *OsNAR2.1* (621 bp) was amplified by PCR and subcloned into the pSAT6-EGFP-C1 vector. The PCR primers with BamHI and EcoRI sites were listed in Supporting Information Table S4. After PI-PspI digestion of pSAT6-EGFP-NAR2.1, the fragment was cloned into the PI-PspI site of vector pRCS2-ocs-nptII in the antisense orientation and confirmed by gene sequencing. To construct the expression vector *OsNAR2.1*-GFP (35S:NAR2.1-GFP), the stop codon of *OsNAR2.1* ORF was removed from the PCR fragment (618 bp) before cloning into pSAT6-EGFP-N1 (Tzfira *et al*., 2005), and was subcloned into pRCS2-ocs-nptII vectors. The primers used are listed in Supporting Information Table S4.

### Fluorescence microscopy

Briefly, 0.2 ml of protoplast suspension of rice blade (*c*. 2 ×10 cells) was transfected with DNA from 35S:GFP-NAR2.1 and 35S:NAR2.1-GFP constructs (10 μg each). The transformed cells were incubated for 16 to 18 h at room temperature before examination in protoplast medium (R2S + 0.4 M mannitol, Nelson *et al*., 2007). To mark the PM location, the transfected protoplasts were stained with 5 μg ml^−1^ of the PM-selective dye marker FM4-64 (Tang *et al*., 2012). Protoplasts were observed under a ×60 objective. GFP expression was visualized by confocal laser scanning microscopy (LSM410; Zeiss AG, Oberkochen, Germany) with a 543 nm helium-neon laser and a 488 nm argon laser, and fluorescent images were captured with a SPOT camera (Tang *et al*., 2012).

To confirm the location of the 35S:GFP-NAR2.1 and 35S:NAR2.1-GFP fusions in rice protoplasts, we performed transient expression in leaves of tobacco (*Nicotiana benthamiana*) mediated by *Agrobacterium tumefaciens*. The combination of the Agrobacterium EHA105 bacterial cells with the indicated constructs above were cultured to an OD_600_ of 1.0 and then infiltrated into leaves of tobacco plants. Infiltrated tobacco plants were incubated for another 3–4 d before fluorescence observation (Liu *et al*., 2012). GFP fluorescence in the cells was analyzed with a 488 nm argon laser using a confocal laser-scanning microscope LSM410 (Zeiss AG).

For BiFC assays, EYFP fluorescence was visualized with a laser wavelength of 525 nm using the confocal microscope described earlier at 24 h after protoplast bombardment of the plasmid pair (Citovsky *et al*., 2008).

### Western blotting

Rice seedlings were grown in IRRI nutrient solution for two months (Yan *et al*., 2011). The PMs were separated from the other membranes by aqueous two-phase partitioning according to the method of Yan *et al*. (2011). The microsome (M) fraction and endomembrane (EM) fraction were separated according to the method of Ishikawa *et al*. (2009). All tissue samples were homogenized and lysed in buffer containing 1% Nonidet P-40 and protease inhibitors. Lysates were cleared by centrifugation and protein concentration was measured spectrophotometrically at A594 nm using Bradford reagent (Sigma-Aldrich, Shanghai, China).

Protein (50 μg) of each sample was boiled in gel loading buffer and resolved on 10% SDS-PAGE gels. Reactive proteins were detected with secondary antibodies including anti-OsNRT2.3a (1:500 dilution, Tang *et al*., 2012), anti-OsNAR2.1 (1:1000 dilution, Yan *et al*., 2011), anti-PIP1 (plasma membrane aquaporin), anti-Bip (ER lumen protein) and anti-V-ATPase (vacuolar H-ATPase subunit a) (1:2000 dilution; Santa Cruz Biotechnology, Santa Cruz, CA, USA, Ishikawa *et al*., 2009). Bands were detected using Kodak film (Rochester, NY, USA).

## Results

### Membrane yeast two-hybrid interactions between OsNAR2.1 and OsNRT2.3a

**Figure 1:**
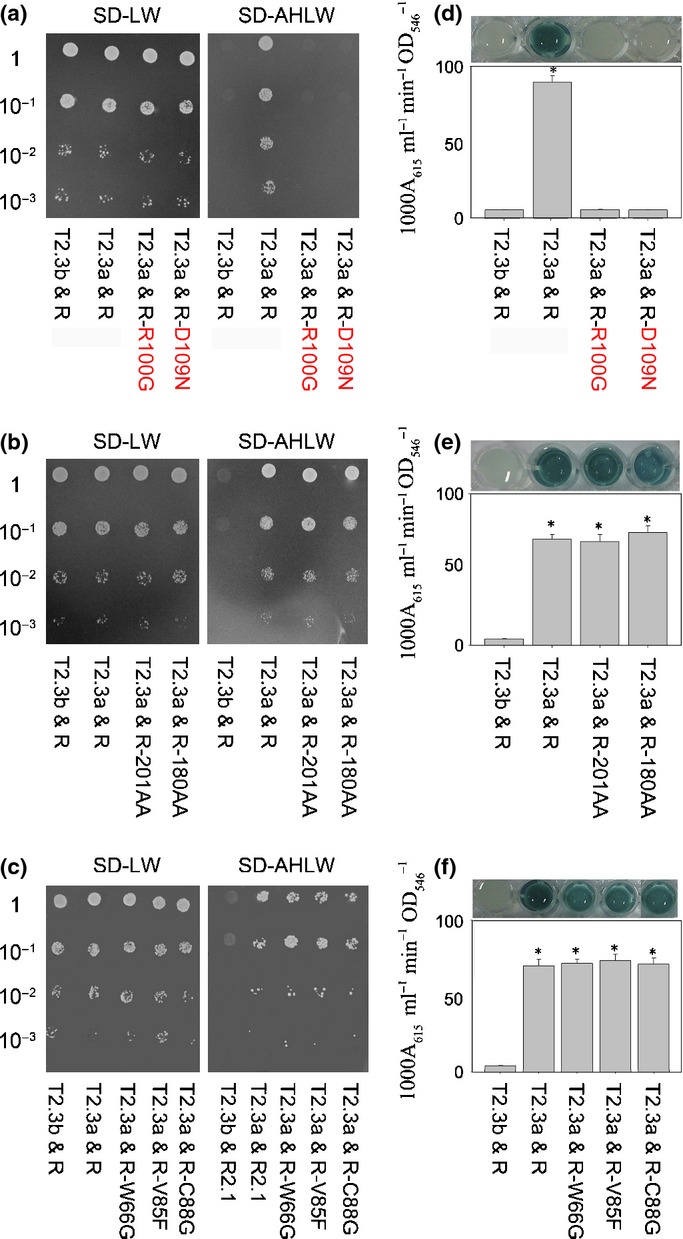
Testing the interaction of OsNAR2.1 mutations with OsNRT2.3a using the DUAL membrane pairwise interaction kit with HIS3, ADE2 and lacZ as reporter genes. Yeast strain NMY51 carrying OsNRT2.3b (T2.3b) in the pPR3-N vector as prey, OsNAR2.1 (R) in the pBT3-C vector as bait and co-expression of T2.3b & R as the negative gene control for membrane protein interactions; and OsNRT2.3a (T2.3a) in the pPR3-N vector as prey, OsNAR2.1 (R) in the pBT3-C vector as bait and co-expression of T2.3a & R as a positive gene control for membrane protein interactions (Yan *et al*., 2011). (a–c) Cells grown on selective control SD-LW block (without Leu and Trp) medium or SD-AHLW block (without Ade, His, Leu and Trp); (d–f) β-galactosidase activity assay for quantification of the interaction strength. For a detailed description of each figure, for example in (a), SD-LW block rows 1–4 show T2.3b & R, T2.3a & R, T2.3a & R-R100G and T2.3a & R-D109N, respectively; in the SD-AHLW block, yeast growth was in the same order as in the SD-LW block. *Significant difference at *P* < 0.05 of the same treatments among the different combinations. The values represent the means ± standard deviation of five replicates.

Interactions between OsNAR2.1 and OsNRT2.3a have previously been reported using the split ubiquitin yeast two-hybrid heterologous system, which can be used to detect interactions between membrane proteins (Yan *et al*., 2011). To examine how OsNAR2.1 interacts with OsNRT2.3a, we separately fused OsNAR2.1 C-terminal deletions and OsNAR2.1 point mutations to the Cub (C-terminal half of ubiquitin) and fused the OsNRT2.3a proteins to the Nub (N-terminal half of ubiquitin). If the OsNAR2.1 and OsNRT2.3a interaction resulted in reconstitution of the split ubiquitin (Nub and Cub), the reporter genes (HIS3 and ADE2) would allow the yeast to grow on selective medium (SD-AHLW). It was found that when OsNAR2.1 with a C-terminal deletion or single point mutation of W66G, V85F, C88G, K101F, R144G, A150G, and G158R were separately transformed with OsNRT2.3a, the yeast could still grow on SD-AHLW (Fig. 1b,c, Supporting Information Fig. S2a); however, the yeast transformed with OsNAR2.1 R100G, R100K or D109N and OsNRT2.3a could not grow (Fig. 1a, Supporting Information Fig. S2c,d). The β-galactosidase activity was indicative of a strong interaction between OsNAR2.1 and OsNRT2.3a. Higher activity levels were detected when OsNAR2.1 with C-terminal deletions W66G, V85F, C88G, K101F, R144G, A150G and G158R mutations were separately co-transformed with OsNRT2.3a into the NMY51 yeast strain compared to the transformation of R100 or D109 mutated OsNAR2.1 with OsNRT2.3a (Fig. 1d–f, Supporting Information Fig. S2b,d).

These observations suggested that point mutations R100G or D109N in OsNAR2.1 inhibited the interaction with OsNRT2.3a; therefore, arginine 100 and aspartic acid 109 of OsNAR2.1 may play key roles in the interaction between OsNAR2.1 and OsNRT2.3a.

### Functional assay of OsNAR2.1 and OsNRT2.3a in *Xenopus* oocytes

**Figure 2:**
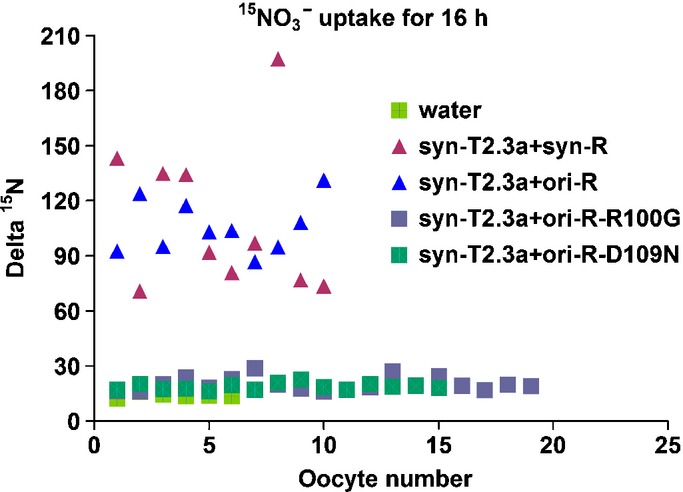
Functional assay of OsNAR2.1 point mutations and OsNRT2.3a for nitrate transport in *Xenopus* oocytes. Uptake of ^15^N–NO_3_^−^ into oocytes injected with water or mixtures, as indicated. Oocytes were incubated for 16 h in modified Barth’s saline containing 0.5 mM ^15^N–NO_3_^−^. Using water as a control; syn-T2.3a + syn-R: oocytes were injected with the mRNA mixture of codon-optimized and synthesized *OsNAR2.1* (see Feng *et al*., 2013) and codon-optimized and synthesized *OsNRT2.3a* (see Feng *et al*., 2013); syn-T2.3a + ori-R: mRNA mixture of rice original *OsNAR2.1* and codon-optimized and synthesized *OsNRT2.3a*; syn-T2.3a + ori-R-R100G: mRNA mixture of rice original *OsNAR2.1* with the D109N mutation and codon-optimized and synthesized *OsNRT2.3a*; syn-T2.3a + ori-R-D109N: mRNA mixture of rice original *OsNAR2.1* with the R100G mutation and optimized, synthesized *OsNRT2.3a*.

We reported that co-injection of both OsNRT2.3a and OsNAR2.1 mRNAs into oocytes resulted in a large nitrate influx (Yan *et al*., 2011). Furthermore, we found that codon optimization of OsNRT2.3a (syn-T2.3a) and OsNAR2.1 (syn-R) cDNAs may improve the functional activity of these proteins expressed in *Xenopus* oocytes (Feng *et al*., 2013). In the present study, we discovered that for the small protein OsNAR2.1, the original gene (ori) had the same expressional function in oocytes as the synthesized gene (syn); that is, oocytes co-injected with syn-OsNRT2.3a (syn-T2.3a) and ori-OsNAR2.1 (ori-R) could reach similar ^15^N accumulation after a 16-h incubation in ^15^N-enriched nitrate as syn-T2.3a and syn-R co-injected oocytes (Fig. 2). Therefore, we used ori-R-R100G and ori-R-D109N to examine the effect of OsNAR2.1 point mutations on nitrate transport activity of OsNRT2.3a in *Xenopus* oocytes. We further analyzed ^15^N-enriched nitrate accumulation of syn-T2.3a + ori-R-R100G or syn-T2.3a + ori-R-D109N co-injected oocytes. The results showed that oocytes co-injected with syn-T2.3a + ori-R-R100G or syn-T2.3a + ori-R-D109N mRNA contained higher levels of 20 Delta ^15^N-nitrate, similar to the concentration in water-injected oocytes (Fig. 2). At the same time, ^15^N-enriched nitrate accumulation in syn-T2.3a + syn-R or syn-T2.3a + ori-R co-injected oocytes could reach 107 Delta ^15^N (Fig. 2). These results indic-ated that the co-injection of syn-T2.3a + ori-R-R100G or syn-T2.3a + ori-R-D109N lost the nitrate uptake function in oocytes. The oocyte ^15^N uptake experiment confirmed the yeast two-hybrid results (Fig. 1), which further indicated that OsNAR2.1 point mutations of R100G and D109N affected the interaction between OsNAR2.1 with OsNRT2.3a (Fig. 2).

### Subcellular localization of OsNAR2.1

**Figure 3:**
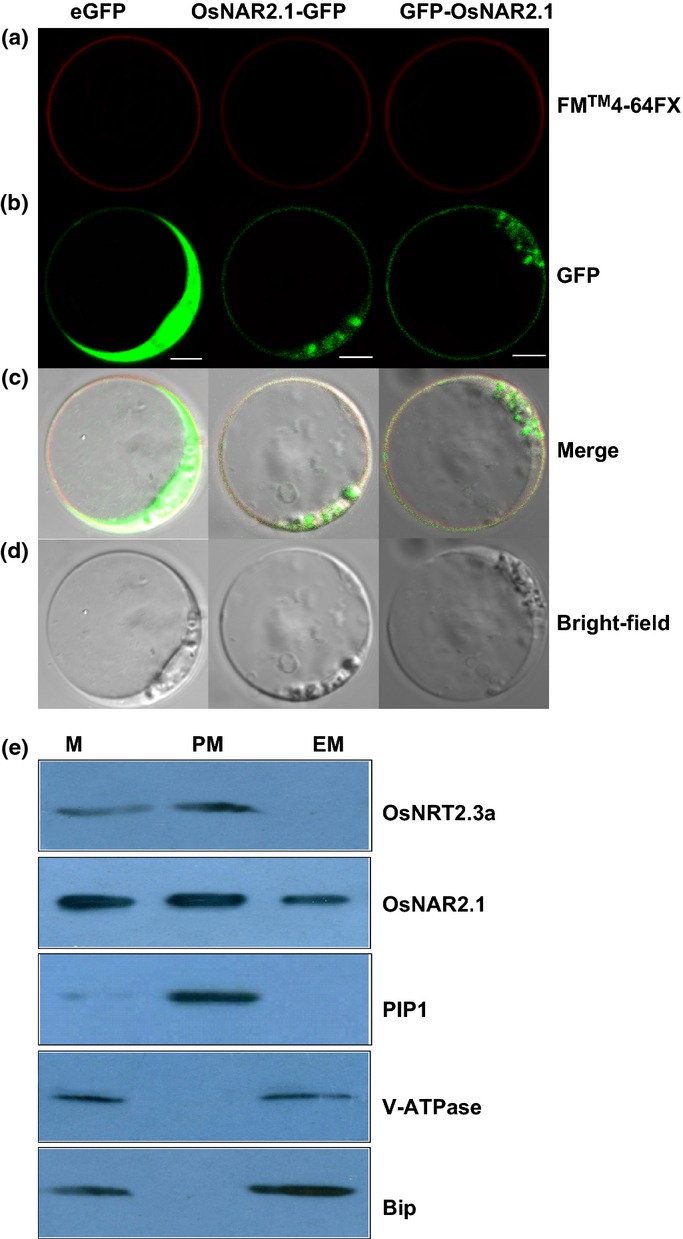
Localization of OsNAR2.1 in rice protoplasts. (a) FM4-64FX dye image; the red fluorescence reflects the position of the plasma membrane (PM). (b) Green fluorescent protein (GFP) fluorescence after expressing NAR2.1-GFP and GFP-NAR2.1 fusion proteins in rice blade protoplasts. (c) Rice protoplasts expressing FM4-64FX (red) and GFP (green) fluorescence. (d) Rice protoplasts in bright field without exciting light. Column 1 shows the protoplasts expressing 35S: GFP was used as a control. Column 2 shows the protoplasts expressing rice OsNAR2.1-GFP fusion protein with FM4-64FX dye. Column 3 shows the protoplasts expressing rice GFP-OsNAR2.1 fusion protein with FM4-64FX dye. FM4-64FX is a membrane-selective fluorescent vital dye. Bars, 10 μm. (e) Immunoblot for OsNRT2.3a, OsNAR2.1, PIP1 (PM marker), V-ATPase (vacuolar marker), and Bip (endoplasmic reticulum (ER) marker) in cell membranes separated from roots of two-month-old rice seedlings. Proteins from microsomes (M), PM and endomembranes (EM) were analyzed on 10% SDS-PAGE gels (50 μg of protein/lane).

We constitutively expressed the fusion protein OsNAR2.1 and GFP using the pSAT6-EGFP-C1 and pSAT6A-EGFP-N1 expression vector (Tzfira *et al*., 2005) to investigate the subcellular localization of OsNAR2.1. We transfected OsNAR2.1-GFP and GFP-OsNAR2.1 fusions into rice blade protoplasts under control of the cauliflower mosaic virus 35S promoter, and GFP expression was determined using confocal microscopy. To further explore the localization of OsNAR2.1, we expressed the OsNAR2.1-GFP and GFP-OsNAR2.1 constructs in tobacco epidermis cells (Supporting Information Fig. S3). GFP signaling demonstrated that the fusion protein was transiently expressed in both PM and cytoplasm whether the GFP was fused to the N- or C-terminus of OsNAR2.1 (Fig. 3a–d, Supporting Information Fig. S3).

To account for the possible effect of GFP on OsNAR2.1 protein localization, we explored the OsNAR2.1 and OsNRT2.3a protein levels in different cellular membranes by Western blotting with specific antibodies. We found that OsNAR2.1 was located in the microsomes (M), which included the endoplasmic reticulum (ER), PM, and EM (mainly including ER) (Fig. 3e), while OsNRT2.3a was located mainly in the PM (Fig. 3e).

### BiFC analysis of OsNRT2.3a and OsNAR2.1

**Figure 4:**
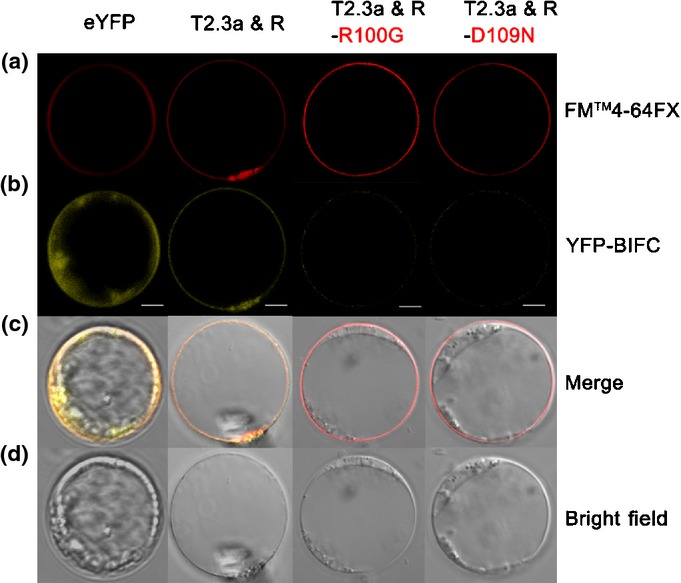
Transient expression of split yellow fluorescent protein (YFP) constructs in rice protoplasts. (a) FM4-64FX dye image; red fluorescence reflects the position of the plasma membrane. (b) YFP fluorescence images: protoplasts were transfected with OsNRT2.3a-cEYFP (T2.3a) and nEYFP-OsNAR2.1 (R). (c) Rice protoplasts expressing EYFP (yellow) with FM4-64FX (red) fluorescence. (d) Rice protoplasts in bright field without exciting light. Column 1, protoplasts expressing YFP co-transfected with PSAT1-nEYFP-C1 and PSAT1-cEYFP-N1 as a control (eYFP). Column 2, protoplasts transfected with OsNRT2.3a-cEYFP (T2.3a) and nEYFP-OsNAR2.1 (R) with FM4-64 dye. Column 3, protoplasts transfected with OsNRT2.3a-cEYFP (T2.3a) and nEYFP-OsNAR2.1 R100G (R-R100G) with FM4-64FX dye. Column 4, protoplasts transfected with OsNRT2.3a-cEYFP (T2.3a) and nEYFP-OsNAR2.1 D109N (R-D109N) with FM4-64FX dye. Bars, 10 μm.

We visualized the interaction between OsNRT2.3a and OsNAR2.1 using transient expression of split EYFP-labeled OsNAR2.1 and OsNRT2.3a in rice blade protoplasts. We constructed C-terminal fusions of EYFP with OsNAR2.1 (nEYFP-OsNAR2.1) and N-terminal fusions of EYFP with OsNRT2.3a (OsNRT2.3a-cEYFP), and transformed protoplasts with both nEYFP-OsNAR2.1 and OsNRT2.3a-cEYFP. YFP was used as a reporter protein because color development occurred only when the two non-fluorescent fragments (nEYFP-OsNAR2.1 and OsNRT2.3a-cEYFP) interacted. Fluorescence was only localized to the PM when protoplasts were transfected with both nEYFP-OsNAR2.1 and OsNRT2.3a-cEYFP (Fig. 4).

Furthermore, we transfected blade protoplasts with nEYFP-OsNAR2.1 R100G and OsNRT2.3a-cEYFP or nEYFP-OsNAR2.1 D109N and OsNRT2.3a-cEYFP, which showed weak or no fluorescence in PM (Fig. 4, Supporting Information Fig. S4), indicative of a poor interaction between the OsNAR2.1 R100G or D109N point mutants with OsNRT2.3a. Thus, the conversion of R100 to G and D109 to N inhibited the interaction of OsNAR2.1 with OsNRT2.3a.

## Discussion

HATS activity in plants is controlled by a two-component NRT2/NAR2 transport system, and NAR2 is a partner protein for the NRT2 to take up nitrate (Feng *et al*., 2011a; Xu *et al*., 2012; references cited therein). Although protein analysis indicated that HvNRT2.1 and HvNAR2 interacted via the C-terminus of HvNRT2.1 and the central region of HvNAR2, the specific localization on the NAR2 loop has not been identified (Ishikawa *et al*., 2009). An *Arabidopsis* mutant (*rnc1*) with a mutation (D105N) in the central region of AtNAR2.1 (AtNRT3.1), which was very important for nitrate uptake, was isolated previously (Kawachi *et al*., 2006). However a complete detailed description of the biochemical activity of this mutation and whether it disrupted the interaction with AtNRT2 partner protein remains unknown. Alignment of NAR sequences among various species showed that this mutation (D105N) occurred in a conserved region of *NAR2.1* among different higher plants, and it is also conserved in OsNAR2.1 (D109) (Supporting Information Fig. S1).

In the present study, point mutations were introduced into the conserved regions of OsNAR2.1 to identify the interacting motifs with NRT2 partners (Supporting Information Fig. S1b). We found that two amino acids, R100 and D109, located in the central region of OsNAR2.1 are essential for the functional interaction with OsNRT2.3a for nitrate transport.

Initially, membrane yeast two-hybrid interactions indicated that point mutations W66G, V85F, C88G, K101F, R144G, A150G and G158R did not affect the interaction between OsNAR2.1 and OsNRT2.3a (Fig. 1c,f, Supporting Information Fig. S2). Removing either 7 AAs or 28 AAs from the C-terminal end of OsNAR2.1 protein did not affect its interaction with OsNRT2.3a, indicating that the C-terminus is not important for the interaction (Fig. 1b,e). However, the R100G, R100K or D109N mutant yeast colonies were unable to grow in SD-AHLW medium, suggesting that the two AAs may affect these interactions (Fig. 1a,d, Supporting Information Fig. S2c,d).

Second, to confirm these yeast results, we conducted an ^15^N uptake experiment in an oocyte system. Co-injection of OsNAR2.1 with a point mutation (R100G or D109N) and OsNRT2.3a mRNA showed no increase in nitrate transport activity when compared to water-injected controls (Fig. 2). These experiments revealed that R100 and D109 in OsNAR2.1 play important roles in the regulation of HATS activity.

Third, we used transient expression of OsNAR2.1-GFP or GFP-OsNAR2.1 in protoplasts of rice blades and tobacco epidermal cells. Interestingly, we found that OsNAR2.1 was transiently expressed not only in the PM but also in the cytoplasm whether the GFP fusion was on the N- or C-terminus of *OsNAR2.1* (Fig. 3, Supporting Information Fig. S3). Using antibodies and Western blot analysis, we found that the expression of OsNAR2.1 was abundant in both the PM and ER (Fig. 3e), confirming the subcellular localization of OsNAR2.1 in rice protoplast (Fig. 3) and transiently transformed tobacco epidermal cells (Supporting Information Fig. S3). HvNAR2 protein was also examined in both microsomes (M) and PM fractions from barley roots by Ishikawa *et al*. (2009), although they found that NAR2 was expressed mainly in the PM. Both Ishikawa *et al*. (2009) and our data based on Western blotting of NAR2.1 indicated that NAR2.1 protein was expressed not only in the PM but also in microsomes (M). Furthermore, OsNAR2.1 was also observed in the EM (mainly ER), which indicated that OsNAR2.1 may remain longer in the ER than HvNAR2.1 during trafficking from the cytosome to the PM.

Because the GFP fusion on the N- and C-terminus of OsNAR2.1 showed a similar expression pattern (Fig. 3, Supporting Information Fig. S3), we suggested that the N- or C-terminus of OsNAR2.1 had no specific sequence for signaling PM location. Based on the GFP expression pattern, we deduced that when we performed BiFC, cYFP-OsNAR2.1 fusion did not damage the original signal for localization of OsNAR2.1. However, as shown in Fig. 4, OsNAR2.1 and OsNRT2.3a co-localized in PM; thus, we explored whether R100 and D109 of OsNAR2.1 are important in targeting OsNAR2.1 and OsNRT2.3a to the PM. As expected, split YFP-labeled OsNRT2.3a and OsNAR2.1 mutants of R100G and D109N did not show any YFP fusion signal in PM (Fig. 4, Supporting Information Fig. S3). The BiFC results suggested that R100 and D109-dependent OsNAR2.1 binding with OsNRT2.3a might be involved in the targeting of OsNAR2.1 to the PM before the partners interact for nitrate transport.

Overall, R100 and D109 in the OsNAR2.1 protein are important not only for establishing HATS activity, but also for co-localization of OsNAR2.1 with OsNRT2.3a at the PM. Since the two amino acids are located in conserved central motifs among known plant NAR2 members (Supporting Information Fig. S1a), it will be interesting to explore whether they perform a similar function for interactions of other NAR2/NRT2 two-component systems. Furthermore, the biological function of these two point mutants requires further characterization *in planta* in future studies.

## Supporting Information

**Fig. S1** Protein sequence alignment and point mutations of OsNAR2.1. **Fig. S2** OsNAR2.1 point mutants (K101F, R144G, A150G, G158R and R100K) interact with OsNRT2.3a in a yeast two-hybrid system. **Fig. S3** Subcellular localization of OsNAR2.1 GFP fusion in tobacco epidermal cells. **Fig. S4** OsNAR2.1 with point mutations fail to co-localize with OsNRT2.3a based on a BiFC analysis. **Table S1** PCR primers used to create mutations of OsNAR2.1 **Table S2** PCR primers used for *Xenopus* oocyte assays **Table S3** PCR primers used for the BiFC assay **Table S4** PCR primers used for transient expression of OsNAR2.1

## References

[b1] Araki R, Hasegawa H (2006). Expression of rice (*Oryza sativa* L.) genes involved in high-affinity nitrate transport during the period of nitrate induction. Breeding Science.

[b2] Cai C, Wang J, Zhu Y, Shen Q, Li B, Tong Y, Li Z (2008). Gene structure and expression of the high-affinity nitrate transport system in rice roots. Journal of Integrative Plant Biology.

[b3] Chopin F, Orsel M, Dorbe M, Chardon F, Truong H, Miller AJ, Krapp A, Daniel-Vedele F (2007). The *Arabidopsis* ATNRT2.7 nitrate transporter controls nitrate content in seeds. Plant Cell.

[b4] Citovsky V, Gafni Y, Tzfira T (2008). Localizing protein–protein interactions by bimolecular fluorescence complementation *in planta*. Methods.

[b5] Feng H, Fan X, Yan M, Liu X, Miller AJ, Xu G (2011a). Multiple roles of nitrate transport accessory protein NAR2 in plants. Plant Signaling & Behavior.

[b6] Feng H, Xia X, Fan X, Xu G, Miller AJ (2013). Optimizing plant transporter expression in *Xenopus* oocytes. Plant Methods.

[b7] Feng H, Yan M, Fan X, Li B, Shen Q, Miller AJ, Xu G (2011b). Spatial expression and regulation of rice high-affinity nitrate transporters by nitrogen and carbon status. Journal of Experimental Botany.

[b8] Goodin MM, Chakrabarty R, Banerjee R, Yelton S, DeBolt S (2007). New gateways to discovery. Plant Physiology.

[b9] Ishikawa S, Ito Y, Sato Y, Fukaya Y, Takahashi M, Morikawa H, Ohtake N, Ohyama T, Sueyoshi K (2009). Two-component high-affinity nitrate transport system in barley: membrane localization, protein expression in roots and a direct protein–protein interaction. Plant Biotechnology.

[b10] Kawachi T, Sunaga Y, Ebato M, Hatanaka T, Harada H (2006). Repression of nitrate uptake by replacement of Asp105 by asparagine in AtNRT3.1 in *Arabidopsis thaliana* L. Plant Cell Physiology.

[b11] Kotur Z, Mackenzie N, Ramesh S, Tyerman SD, Kaiser BN, Glass ADM (2012). Nitrate transport capacity of the *Arabidopsis thaliana* NRT2 family members and their interactions with AtNAR2.1. New Phytologist.

[b12] Liu TY, Huang TK, Tseng CY, Lai YS, Lin SI, Lin WY, Chen JW, Chiou T-J (2012). PHO2-dependent degradation of PHO1 modulates phosphate homeostasis in *Arabidopsis*. Plant Cell.

[b13] Miller AJ, Fan X, Orsel M, Smith SJ, Wells DM (2007). Nitrate transport and signaling. Journal of Experimental Botany.

[b14] Nelson BK, Cai X, Nebenführ A (2007). A multicolored set of *in vivo* organelle markers for co-localization studies in *Arabidopsis* and other plants. Plant Journal.

[b15] Orsel M, Chopin F, Leleu O, Smith SJ, Krapp A, Daniel-Vedele F, Miller AJ (2006). Characterization of a two-component high-affinity nitrate uptake system in *Arabidopsis* physiology and protein–protein interaction. Plant Physiology.

[b16] Plett D, Toubia J, Garnett T, Tester M, Kaiser BN, Baumann U (2010). Dichotomy in the NRT gene families of dicots and grass species. PLoS One.

[b17] Quesada A, Galván A, Fernández E (1994). Identification of nitrate transporter genes in *Chlamydomonas reinhardtii*. Plant Journal.

[b18] Tang Z, Fan X, Li Q, Feng H, Miller AJ, Shen Q, Xu G (2012). Knock-down of a rice stelar nitrate transporter alters long-distance translocation but not root influx. Plant Physiology.

[b19] Tong Y, Zhou JJ, Li Z, Miller AJ (2005). A two-component high-affinity nitrate uptake system in barley. Plant Journal.

[b20] Tzfira T, Tian GW, Lacroix B, Vyas S, Li J, Leitner-Dagan Y, Krichevsky A, Taylor T, Vainstein A, Citovsky V (2005). pSAT vectors: a modular series of plasmids for autofluorescent protein tagging and expression of multiple genes in plants. Plant Molecular Biology.

[b21] Wirth J, Chopin F, Santoni V, Viennois G, Tillard P, Krapp A, Lejay L, Daniel-Vedele F, Gojon A (2007). Regulation of root nitrate uptake at the NRT2.1 protein level in *Arabidopsis thaliana*. Journal of Biological Chemistry.

[b22] Xu G, Fan X, Miller AJ (2012). Plant nitrogen assimilation and use efficiency. Annual Review of Plant Biology.

[b23] Yan M, Fan X, Feng H, Miller AJ, Shen Q, Xu G (2011). Rice OsNAR2.1 interacts with OsNRT2.1, OsNRT2.2 and OsNRT2.3a nitrate transporters to provide uptake over high and low concentration ranges. Plant, Cell & Environment.

[b24] Yong Z, Kotur Z, Glass ADM (2010). Characterization of an intact two-component high-affinity nitrate transporter from *Arabidopsis* roots. Plant Journal.

[b25] Zhou JJ, Fernandez E, Galvan A, Miller AJ (2000). A high affinity nitrate transport system from *Chlamydomonas* requires two gene products. FEBS Letters.

