# Supplementary material for: Identification and functional assay of the interaction motifs in the partner protein OsNAR2.1 of the two-component system for high-affinity nitrate transport
Source: New Phytol. 2014 Aug 7;204(1):74–80. doi: 10.1111/nph.12986 (PMC4232926; doi:10.1111/nph.12986)
Supplement: Supplementary file 1 — Fig. S1 Protein sequence alignment and point mutations of OsNAR2.1. Fig. S2 OsNAR2.1 point mutants (K101F, R144G, A150G, G158R and R100K) interact with OsNRT2.3a in a yeast two-hybrid system. Fig. S3 Subcellular localization of OsNAR2.1 GFP fusion in tobacco epidermal cells. Fig. S4 OsNAR2.1 with point mutations fail to co-localize with OsNRT2.3a based on a BiFC analysis. Table S1 PCR primers used to create mutations of OsNAR2.1 Table S2 PCR primers used for Xenopus oocyte assays Table S3 PCR primers used for the BiFC assay Table S4 PCR primers used for transient expression of OsNAR2.1 [file nph0204-0074-sd1.doc]

**Identification and functional assay of the interaction motifs in the partner protein OsNAR2.1 of the two component system for high affinity nitrate transport**

Xiaoqin Liu, Daimin Huang, Jinyuan Tao, Anthony J. Miller, Xiaorong Fan and Guohua Xu

**Supporting Information**

**Fig. S1** *Protein sequence alignment and point mutations of OsNAR2.1.*

**Fig. S2** *OsNAR2.1 point mutants (K101F, R144G, A150G, G158R and R100K) interact with OsNRT2.3a in a yeast two-hybrid system.*

**Fig. S3** *Subcellular localization of OsNAR2.1 GFP fusion in tobacco epidermal cells.*

**Fig. S4** *OsNAR2.1 with point mutations fail to co-localize with OsNRT2.3a based on a BiFC analysis.*

**Table S1** *PCR primers used to create mutations of OsNAR2.1.*

**Table S2** *PCR primers used for Xenopus oocyte assays.*

**Table S3** *PCR primers used for the BiFC assay.*

**Table S4** *PCR primers used for transient expression of OsNAR2.1.*

**Fig. S1** *Protein sequence alignment and point mutations of OsNAR2.1.*

1. Alignment of nine Nar2 family members containing a homologous conserved motif of the identified peptides corresponding to OsNAR2.1, AtNAR2.1, HvNAR2.3, CrNAR2, OsNAR2.2, HvNAR2.1, HvNAR2.2, TaNAR2.1, and ZmNAR2.1 proteins. The blue background shows highly conserved amino acids, while the pink shows the second most highly conserved. (b) Comparison of the partial OsNAR2.1 sequences and OsNAR2.1 point mutants. The sequences between AA 66 and AA 158 were homologous and conserved within the collection of NAR2 proteins, where we generated the following point mutations: W66G, V85F, C88G, K101F, R144G, A150G, G158R, R100W and D109N. Amino acid sequences of the OsNAR2.1 and the OsNAR2.1 point mutants (lower sequence) are aligned: R100W (or R100K) and D109N with the red style are key mutations blocking the protein interaction in the following experiment.


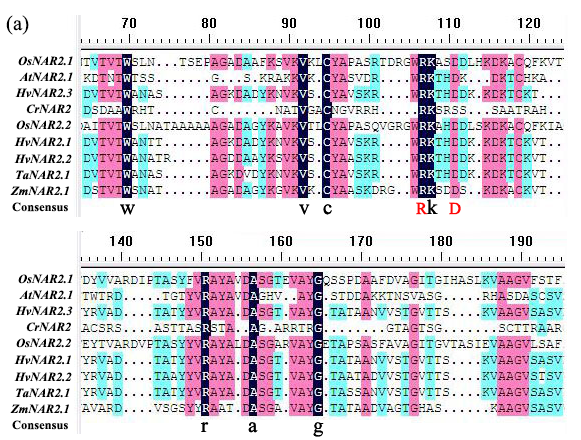


(b)

|  | **M A W V C R K D R A G** |
| --- | --- |
| ***OsNAR2.1*** | ATGGCG...TGG...GTG...TGC...CGC.............AAG...GAC...CGC...GCG...GGG... |
| **Mutations** | ATGGCG...GGG...TTC...GGC...GGC/AAG...TTC....AAC...GGC...GGC...CGG... |
|  | **M A G F G G /K F N G G R** |
|  | 1 2 66 85 88 **100** 101 **109** 144 150 158 |

**Fig. S2** *OsNAR2.1 point mutants (K101F, R144G, A150G, G158R and R100K) interact with OsNRT2.3a in a yeast two-hybrid system.*

Yeast strain NMY51 carried OsNRT2.3b (T2.3b) in the pPR3-N vector as prey, OsNAR2.1 (R) in the pBT3-C vector as bait and co-expression of T2.3b & R as the negative gene control for membrane protein interactions; OsNRT2.3a (T2.3a) in the pPR3-N vector as prey, OsNAR2.1 (R) in the pBT3-C vector as bait and co-expression of T2.3a & R as a positive gene control for the membrane protein interaction (Yan *et al.*, 2011). (a, c) Cells grown on selective control SD-LW block (without Leu and Trp) medium or SD-AHLW block (without Ade, His, Leu and Trp); (b, d) β-galactosidase activity assay for quantification of interaction strength. For a detailed description of each figure; for example in Fig. S2a, the SD-LW block rows 1 to 6 represent T2.3b & R, T2.3a &R, T2.3a & R-K101F, T2.3a & R-R144G, T2.3a & R-A150G and T2.3a & R-G158R, respectively; in SD-AHLW block, yeast growth was in the same order as in the SD-LW block. * Significant difference at P < 0.05 of the same treatments among the different combinations. The values represent means ± SD of five replicates.
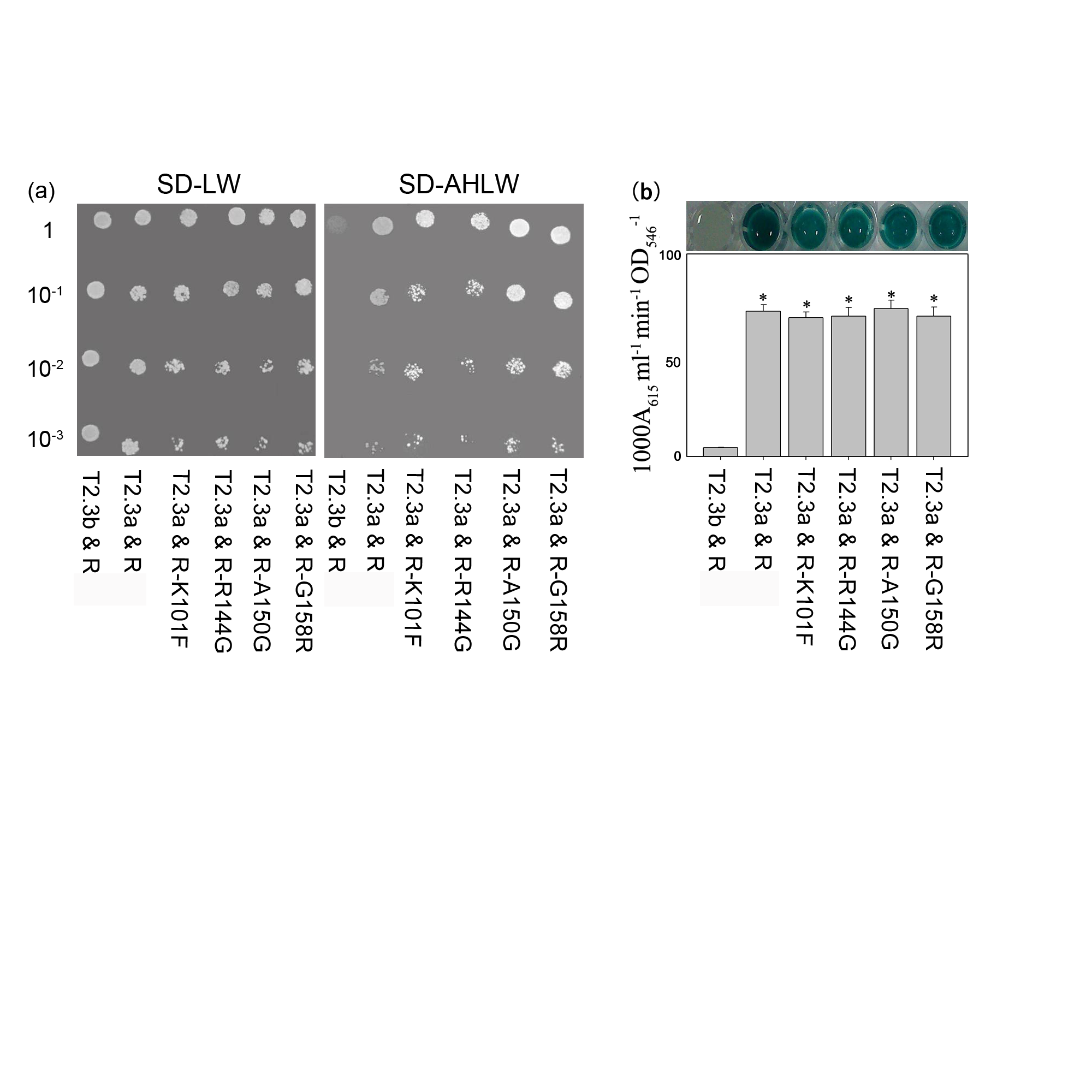


**
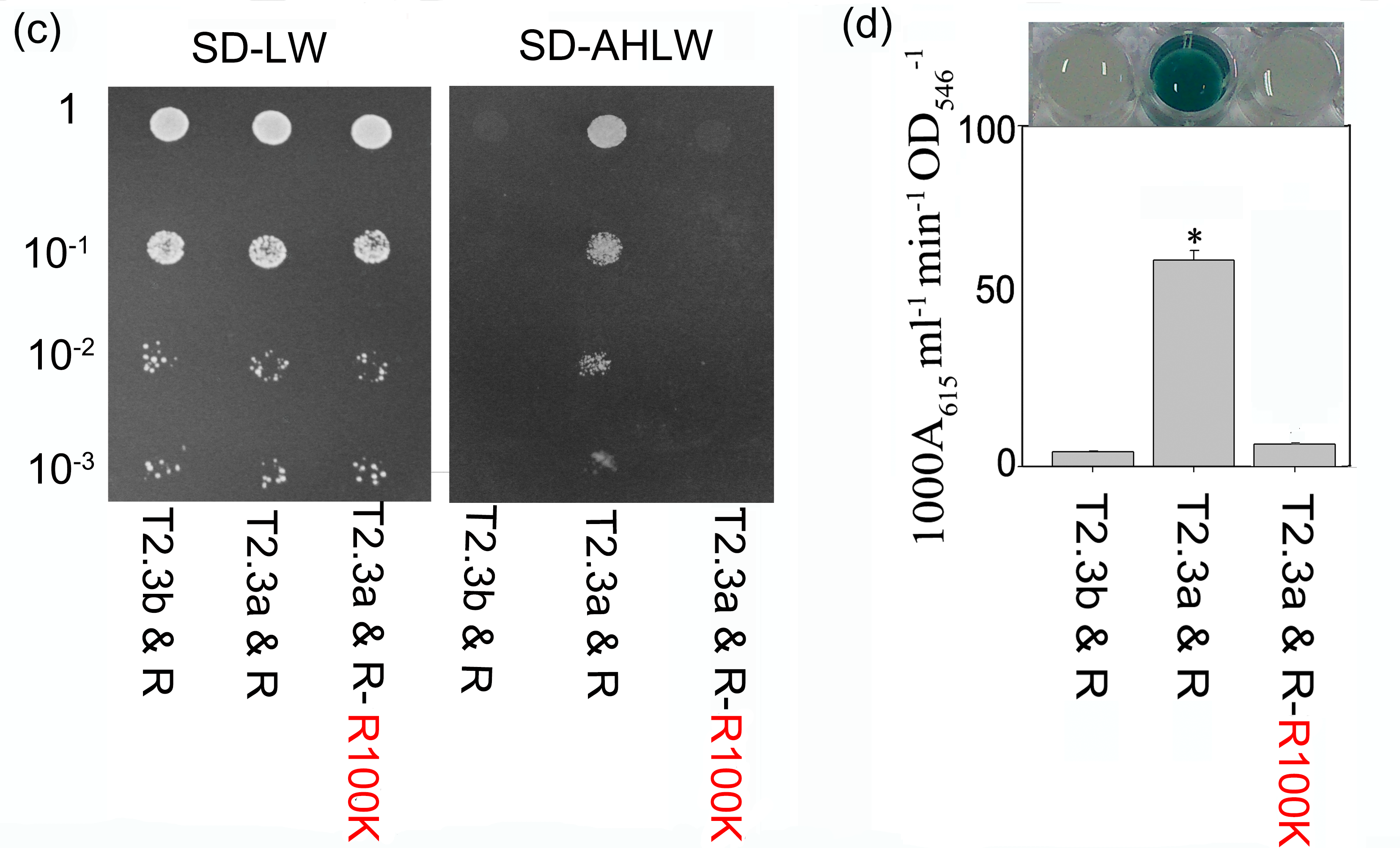
**

**Reference**

**Yan M, Fan X, Feng H, Miller AJ, Shen Q, Xu G.** **2011.** Rice OsNAR2.1 interacts with OsNRT2.1, OsNRT2.2 and OsNRT2.3a nitrate transporters to provide uptake over high and low concentration ranges. *Plant, Cell & Environment* **34**: 1360–1372.

**Fig. S3** *Subcellular localization of OsNAR2.1 GFP fusion in tobacco epidermal cells.*

(a) Micrographs of the NAR2.1-GFP and GFP-NAR2.1 fusion proteins in tobacco (*N. benthamiana*) epidermal cells. (a) Cells expressing GFP in the exciting light. (b) Overlap cell of the GFP (green) and a bright field. (c) The tobacco epidermal cells without exciting light in bright field. Column 1 shows the cells expressing GFP used as a control. Column 2 shows the cells expressing rice OsNAR2.1-GFP fusion protein. Column 3 shows the cells expressing GFP-OsNAR2.1 fusion protein. Red arrow: PM; Purple arrow: EM. Bars, 20 μm.


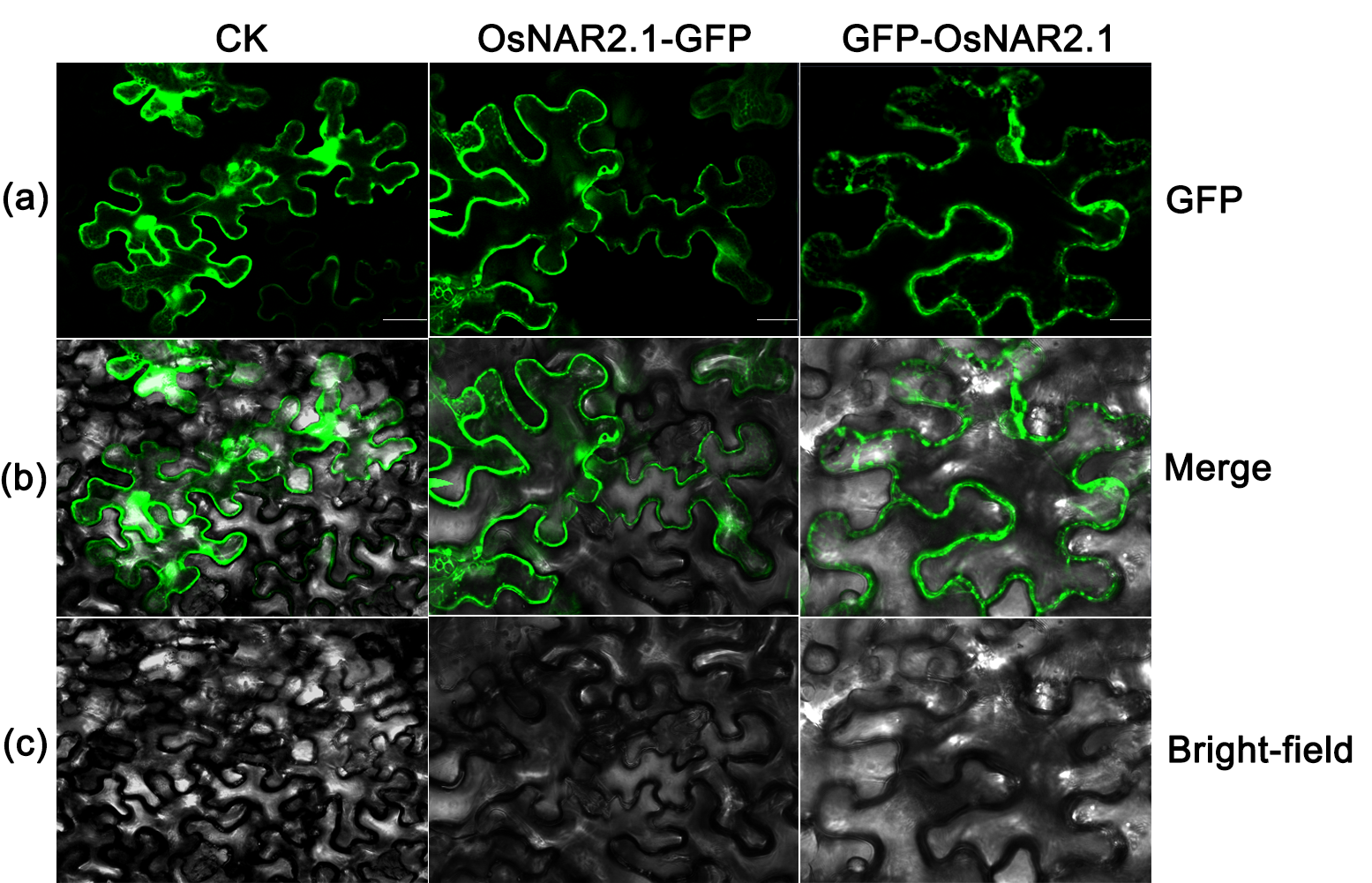


**Fig. S4** *OsNAR2.1 with point mutations fail to co-localize with OsNRT2.3a based on a BiFC analysis.*

Two alternative interaction partners, OsNRT2.3a and OsNAR2.1 R100G or OsNAR2.1 D109N, were fused to fragments of different fluorescent proteins. (a) FM4-64FX dye image: the red fluorescence reflects the position of the plasma membrane. (b) EYFP fluorescence images: protoplasts were transfected with nEYFP-OsNAR2.1 point mutations and OsNRT2.3a-cEYFP. (c) The rice protoplasts expressing EYFP (yellow) with FM4-64FX (red) fluorescence. (d) The rice protoplasts in bright field without exciting light. Column 1 shows the protoplast expressing YFP co-transfected with PSAT1-nEYFP-C1 and PSAT1-cEYFP-N1 as a control. Columns 2 and 3 show the protoplasts transfected with nEYFP-OsNAR2.1 R100G and OsNRT2.3a-cEYFP with FM4-64 dye. Columns 4 and 5 show the protoplasts transfected with nEYFP-OsNAR2.1 D109N and OsNRT2.3a-cEYFP with FM4-64 dye. Scale bars = 10 μm. T, OsNRT; R, OsNAR2.1.


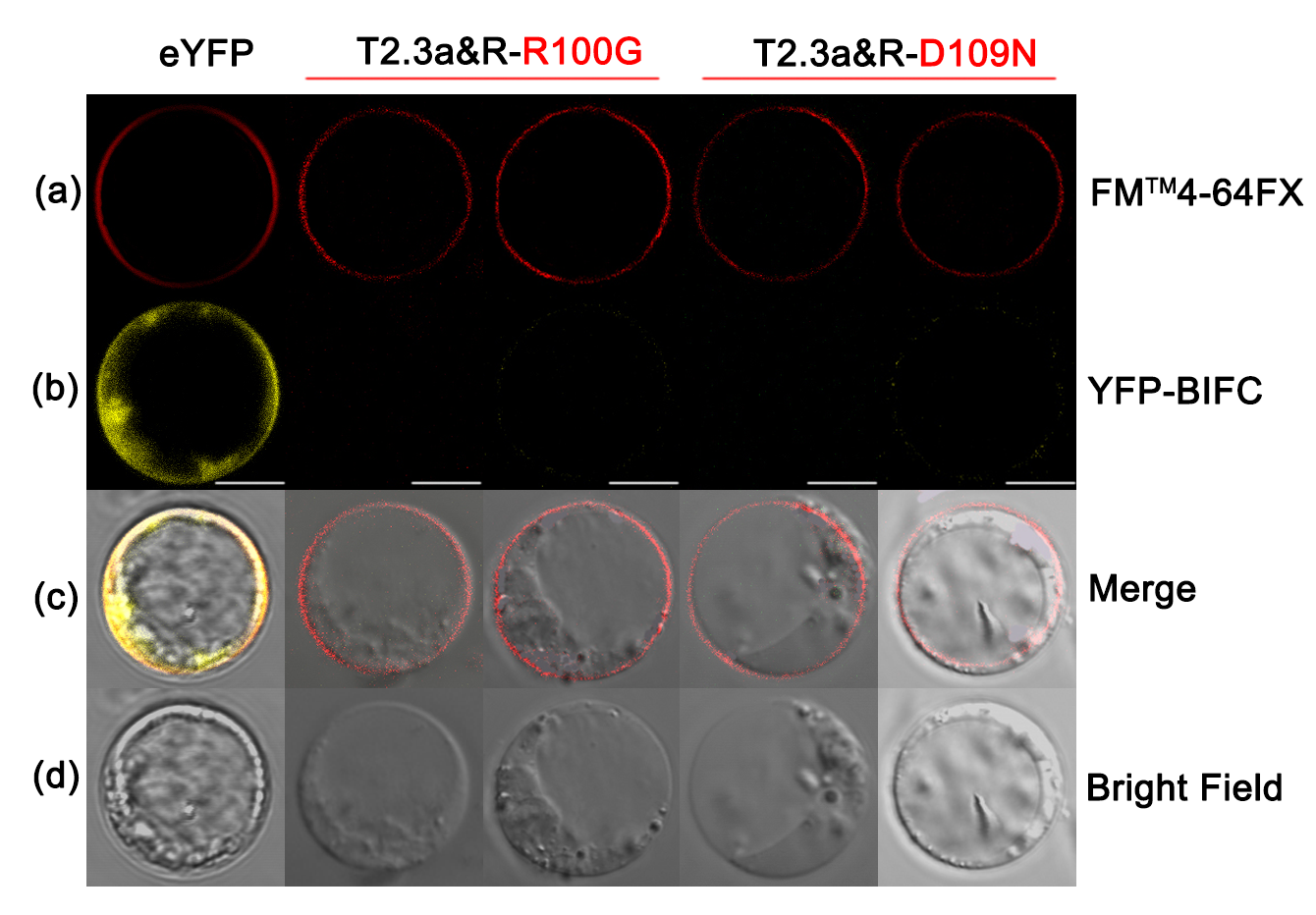


***Table S1*** *PCR primers used to create mutations of OsNAR2.1.*

| **Construct** | **Primer Sequence (5'–3')** | |
| --- | --- | --- |
| W66G | Forward | GAACACGGTGACGGTGACGGGGTCGCTGAACACGTCGGAG |
| W66G | Reverse | CTCCGACGTGTTCAGCGACCCCGTCACCGTCACCGTGTTC |
| V85F | Forward | GGCGTTCAAGAGCGTGAAGTTCAAGCTGTGCTACGCGCCG |
| V85F | Reverse | CTCGCCGGCGCGTAGCACAGCTTGAACTTCACGCTCTTGAACGCCG |
| C88G | Forward | GAGCGTGAAGGTGAAGCTGCGCTACGCGCCGGCGAGCC |
| C88G | Reverse | GGCTCGCCGGCGCGTAGCGCAGCTTCACCTTCACGCTC |
| R100G | Forward | CCGGACGGACCGCGGGTGGGGCAAGGCCTCCGACGACCTG |
| R100G | Reverse | CAGGTCGTCGGAGGCCTTGCCCCACCCGCGGTCCGTCC |
| R100K | Forward | CCGGACGGACCGCGGGTGGGGCAAGGCCTCCGACGACCTG |
| R100K | Reverse | CAGGTCGTCGGAGGCCTTCTTCCACCCGCGGTCCGTCC |
| K101F | Forward | GGACGGACCGCGGGTGGCGCGCGGCCTCCGACGACCTGCAC |
| K101F | Reverse | GTGCAGGTCGTCGGAGGCCGCGCGCCACCCGCGGTCCGTCC |
| D109N | Forward | CCTCCGACGACCTGCACAAGAACAAGGCGTGCCAGTTCAAG |
| D109N | Reverse | CTTGAACTGGCACGCCTTGTTCTTGTGCAGGTCGTCGGAGG |
| R144G | Forward | GTGACCTTGAACTGGCACGCGCCGTCCTTGTGCAGGTCGTCGG |
| R144G | Reverse | CGACGGCGTCCTACTTCGTGGGCGCCTACGCGGTGGACGCG |
| A150G | Forward | CGCGTCCACCGCGTAGGCGCCCACGAAGTAGGACGCCGTCG |
| A150G | Reverse | CAGCTTCACCTTCACGCTCGCGAACGCCGCGTCGGCGCCC |
| G158R | Forward | TGCGCGCCTACGCGGTGGACAGCTCCGGCACGGAGGTGGCCTAC |
| G158R | Reverse | GCCCCCTTGTCCTTCTTGCGCTTCTGGACGACGAAGAAGAAGGCG |
| Deletion  (180AA) | Forward | TAATGAATTCATGGCGAGGCTAGCCGGCGT |
| Deletion  (180AA) | Reverse | TAATGGATCCTGAGGGAGGCGTGGATGCCGG |
| Deletion  (201AA) | Forward | TAATGAATTCATGGCGAGGCTAGCCGGCGT |
| Deletion  (201AA) | Reverse | GCAGGGATCCCGACGACGAAGAAGAAGGCGAGC |

***Table S2*** *PCR primers used for Xenopus oocyte assays.*

| **Construct** | **Primer Sequence (5'--3')** | |
| --- | --- | --- |
| pT7Ts-*NAR2.1* | Forward | AATCAGATCTCAATGGCGAGGCTAGCC |
| pT7Ts-*NAR2.1* | Reverse | CAGAACTAGTCGATCTACTTGTCCTTC |
| pT7Ts-*NRT2.3a* | Forward | AATCAGATCTTTGGAGCTCCACCGC |
| pT7Ts-*NRT2.3a* | Reverse | CAGAACTAGTCCCCCCCTCGAAGG |

***Table S3*** *PCR primers used for the BiFC assay.*

| **Construct** | **Primer Sequence (5'--3')** | |
| --- | --- | --- |
| nEYFP-*NAR2.1* | Forward | TAATCCATGGATGGCGAGGCTAGCCGGCGT |
| nEYFP-*NAR2.1* | Reverse | GCAGAGATCTCTTGTCCTTCTTGCGCTTCTC |
| *NRT2.3a-*cEYFP | Forward | taatccatggATGGAGGCTAAGCCGGTG |
| *NRT2.3a*-cEYFP | Reverse | ttaaagatctTCACACCCCGGCCGGCGACGCG |

***Table S4*** *PCR primers used for transient expression of OsNAR2.1*.

| **Construct** | **Primer Sequence (5'--3')** | |
| --- | --- | --- |
| NAR-GFP | Forward | taatgaattcATGGCGAGGCTAGCCGGCGT |
| NAR-GFP | Reverse | gcagggatccACTTGTCCTTCTTGCGCTTCTC |
| GFP-NAR | Forward | taatgaattcAATGGCGAGGCTAGCCGGCGT |
| GFP-NAR | Reverse | gcagggatccCTACTTGTCCTTCTTGCGCTTCTC |
